# Supplementary material for: Derivation and validation of the J-CTO extension score for pre-procedural prediction of major adverse cardiac and cerebrovascular events in patients with chronic total occlusions
Source: PLoS One. 2020 Sep 11;15(9):e0238640. doi: 10.1371/journal.pone.0238640 (PMC7485776; doi:10.1371/journal.pone.0238640)
Supplement: S1 Study protocol — (DOCX) [file pone.0238640.s001.docx]

Retrograde summit registry: Japanese multicenter registry of percutaneous coronary intervention for coronary chronic total occlusion,.

1. Research Background

The ratio of Percutaneous Coronary Intervention (PCI) for chronic total occlusion (CTO) is estimated to be performed in about 10% for patients with ischemic heart disease. However, there is no data that reveals the current status of CTO-PCI, such as the number of cases performed annually, the success rate of the procedure, the complication rate, etc. in Japan.

2. Purpose of research

To reveal the current status of CTO-PCI in Japan, collecting and analyzing data, making academic progress, clarifying future issues, and examining methods for solution of those issues. In addition, by comparing data in cooperation with other countries / regions, it can contribute to promoting the spread of PCI to CTOs and improving the success rate from a global perspective.

3. Types of study design

Prospective registry observational study

Build a CTO-PCI registry using the Internet database system. At the facilities participating in the Retrograde Summit Study Group, all CTO-PCI cases will be registered, required submit items will be registered in this database, and analysis items such as the total number of cases will be analyzed every year.

4. Research period

Target registration period: Registration starts on January 1, 2012

Subject tracking period: 3 years

5. Selection of target person

All cases of CTO-PCI except the case enrolled in the registry of the Japan Chronic Complete Occlusion Intervention Experts.

6. Observation items (attached case report)

Basic information ①: Entry No. (number automatically assigned), date of birth / age (*), gender (*), height, weight, BMI, medical history / history

Basic information ②: Clinical symptoms, degree of angina, NYHA classification, preoperative examination information, left heart function, myocardial viability

Lesion background: target blood vessel, lesion information, PCI history for target lesion

Basic procedure information: procedure background, procedure success, detailed treatment method information, intraoperative complications

Adverse events: occurrence of MACCE (death, MI, stroke, emergency CABG, emergency PCI) and other adverse events

Chronic phase information: Clinical follow-up, imaging follow-up information

* Personal information (*) can be rejected at the discretion of the ethics committee of each facility

7. Expected adverse events

Same as the adverse event that expected in general PCI case.

8. Main analysis items

Background: Total number of CTO-PCI cases, gender ratio, lesion branch distribution, past PCI history for the lesion, procedure distribution, success rate, complication rate, breakdown of complications

Lesion background: distribution of devices used, distribution of successful procedures, long term patency

9. Protection of personal information

Patient identification in the Retrograde Summit database is performed using only the patient registration number, and careful attention should be paid to the protection of confidential patient information. Register confidential patient information (date of birth, gender, etc.) is left to the judgment of each facility. Also, pay attention to the security of the data at each facility, and do not use the input data for any purpose other than the registry. In publishing the results obtained by this registry, we will give due consideration to the protection of patient personal information. In addition, the doctor and their institution should cooperate with the Registry in browsing and disclosing materials.

10. Ethics of this study

The Registry is implemented pursuant to the Declaration of Helsinki (1980), subject to the consent of the patient or family prior to the Registry. The informed consent is granted to the patient (or his / her family) by the physician, and patients are able to withdraw participation at any time after consent. The human rights of the subject of patients are protected. Obtain consent after fully explaining the necessary items, etc., and sign the consent form.

11. Conflicts of interest in this study

Before any publication and submission, confirmed there is no any possible interests that may affect the Registry's results and interpretation.

12. Compensation for health damage

Compensation related with this registry is covered at each facility and shall be made only when liability is required by law.

13. Treatment costs

The examinations and treatments related to this registry are within the scope of all routine clinical practice, and will be handled under the insurance medical care.

14. Principal Investigator

Maoto Habara (Toyohashi Heart Center)

15. Research organization

Participating facilities: Retrograde Summit study group participating facilities 66 facilities (as of December 25, 2014)

Secretariat: Retrograde Summit Secretariat (email: retrograde.approach@gmail.com)
