# Supplementary material for: Derivation and validation of the J-CTO extension score for pre-procedural prediction of major adverse cardiac and cerebrovascular events in patients with chronic total occlusions
Source: PLoS One. 2020 Sep 11;15(9):e0238640. doi: 10.1371/journal.pone.0238640 (PMC7485776; doi:10.1371/journal.pone.0238640)
Supplement: Attachment — Submitted filename: Response to Reviewers.docx [file pone.0238640.s002.docx]

May 3^rd^, 2020

Dr. Yoshiaki Taniyama

Academic Editor of *PLOS ONE*

Dear Dr. Yoshiaki Taniyama:

Ref. No.:  PONE-D-20-00743
Title: Derivation and Validation of the PJ-CTO Score for Pre-Procedural Prediction of Major Coronary and Cerebrovascular Events in Patients with Chronic Total Occlusions.

I, along with my coauthors, would like to re-submit the attached manuscript as an Original Article.

The manuscript has been carefully rechecked and appropriate changes have been made in accordance with the reviewers’ suggestions. The responses to their comments have been prepared and attached herewith.

We thank you and the reviewers for your thoughtful suggestions and insights, which have enriched the manuscript and produced a more balanced and better account of the research. We hope that the revised manuscript is now suitable for publication in your esteemed PLOS ONE:

We look forward to hearing from you at your earliest convenience.

Sincerely,

Soichiro Ebisawa, MD, PhD

Department of Cardiovascular Medicine, Shinshu University School of Medicine

3-1-1 Asahi, Matsumoto-shi 390-8621, Japan

Tel: +81-263-37-3486/Fax: +81-263-37-3489

Email: [ebisawa@shinshu-u.ac.jp](mailto:ebisawa@shinshu-u.ac.jp)

Comments from the editors and reviewers:

Reviewer #1: I have read with great interest the manuscript entitled “Derivation and validation of the J-CTO extension score for pre-procedural prediction of major adverse cardiac and cerebrovascular events in patients with chronic total occlusions”. The paper makes a significant contribution to the understanding of potential risk factors affecting the long-term prognosis of patients undergoing PCI for CTO. The article is well written, comprises a large number of patients treated at experienced Japanese centres, and a robust methodology provides a new perspective on the problem. Yet, a couple of issues should be elaborated or explained.

Response

We would like to express our appreciation to the Reviewer for his/her insightful comments, which have helped us to improve the report significantly.

Comment 1
The inclusion/exclusion criteria seem unclear since the authors claim that they excluded from the baseline cohort (4909 patients) 770 subjects due to the lack of one-year follow-up data. From the remaining 4139 patients, they excluded another 2230, again because of the lack of one-year follow-up;

Response

Indeed, I thought it was unclear and difficult to understand the meaning of sentence. It means 770 patients who underwent procedures in institutions that did not join collect follow-up one-year data, and I excluded such institution’s data at first. However, 2230 patients still had no data of one-year follow up and should be excluded.

According to the reviewer’s comment, we change the sentences as “did not join to collect one-year follow-up data” in line 16 page 6.

Comment 2
Very few data on PCI techniques were presented in the document. The authors should submit at least the percentage of patients revascularized with antegrade and retrograde technique. Also, it is desirable to mention if the PCI technique had any influence on the clinical outcomes;

Response

The reviewer raise an important point.

According to the reviewer’s comment, we add the sentences “Regarding with technical issue, retrograde approach was performed in 606 cases (31.7%), however this procedure was not associated with both initial success (p=0.569) and incidence of one-year MACCE (p=0.368)” in line 11 page 10.

Comment 3
One of the most surprising observations is the fact that the initial success of the procedure was not associated with the incidence of one-year MACCE. The authors just mention this very casually; I believe they should present more data on this, at least respective percentages in Table 2;

Response

CTO-PCI has been improved latest 10 years, however initial success might not necessarily reflect the long-term outcome. We think this comment is very important in this field, and we should establish another registry to assess this point.

According to the reviewer’s comment, we add the correlation between initial procedural success and incidence of one-year MACCE in table 2.

Comment 4
Another remarkable finding was that in-stent occlusion lesions were associated with the incidence of MACCE; I believe the authors should elaborate on this observation in the Discussion section;

Response

This comment is also important. Long term outcome of PCI after in stent occlusion case was not favorable. We should assess the indication of revascularization of in stent occlusion lesion.

According to reviewer’s comment, we add the sentence of “In this population, one-year survival, non-fatal myocardial infarction and non-fatal stroke were not different between in-stent occlusion and not in-stent occlusion group, however target vessel revascularization was significantly higher in in-stent occlusion group (p<0.0001)” in line 8 page 23.

Comment 5
Finally, recently The EuroCTO (CASTLE) Score has been published. Would it be possible to analyze its predictive value, just as the authors did for two other scores (PROGRES and CL score?

Response

When, first time, we submit this manuscript, CASTLE score was not established. We also think this score is well-established score in big population data. According to the comment we compare between J-CTO extension score and CASTLE (Figure A), CASTLE score was not associated with one-year MACCE.

Figure A

Only J-CTO extension score was associated with one-year MACCE (AUC=0.647).
