# Supplementary material for: Derivation and validation of the J-CTO extension score for pre-procedural prediction of major adverse cardiac and cerebrovascular events in patients with chronic total occlusions
Source: PLoS One. 2020 Sep 11;15(9):e0238640. doi: 10.1371/journal.pone.0238640 (PMC7485776; doi:10.1371/journal.pone.0238640)
Supplement: Attachment — Submitted filename: Response to Reviewers.docx [file pone.0238640.s003.docx]

July 15th, 2020

Dr. Yoshiaki Taniyama

Academic Editor

*PLOS ONE*

Dear Dr. Yoshiaki Taniyama:

Ref. No.: PONE-D-20-00743
Title: Derivation and Validation of the PJ-CTO Score for Pre-Procedural Prediction of Major Coronary and Cerebrovascular Events in Patients with Chronic Total Occlusions.

I, along with my coauthors, would like to re-submit the attached manuscript as an Original Article in *PLOS ONE*. The manuscript has been carefully rechecked and appropriate changes have been made in accordance with the reviewers’ suggestions. In particular, we have addressed all the reviewer's comments in a point-by-point manner and revisions are indicated in red font in the revised paper. We would like to thank you and the reviewers for your thoughtful suggestions and insights, which have enriched the manuscript and produced a more balanced and better account of the research.

We hope that the revised manuscript is viewed favorably. We look forward to hearing from you.

Sincerely,

Soichiro Ebisawa, MD, PhD

Department of Cardiovascular Medicine, Shinshu University School of Medicine

3-1-1 Asahi, Matsumoto-shi 390-8621, Japan

Tel: +81-263-37-3486/Fax: +81-263-37-3489

Email: [ebisawa@shinshu-u.ac.jp](mailto:ebisawa@shinshu-u.ac.jp)

**Reviewer #2: PONE-D-20-00743R1: statistical review**

SUMMARY. This paper investigates factors that increase the risk of developing major adverse events (MACCE) among patients with chronic total occlusion who undergo percutaneous coronary intervention (PCI). The statistical analysis is made in two steps. First a battery of logistic regression models is estimated to identify the significant predictors of MACCE. Then the results of the logistic regression are used to define a risk score. I have little to say about the first step: logistic regression is the correct methodology to identify risk factors and it is appropriately developed in the paper. I have however some concerns about the second step (see major issues below). I also list below some specific points that should be addressed.

MAJOR ISSUES

1) The multivariate logistic regression estimates provided in Table 3 provide an optimal way to estimate the probability of a MACCE, through the formula
Prob(MACCE01)= exp(beta_0+beta_1x_1+ ... \beta_Kx_k)/(1+exp(beta_0+beta_1x_1+ ... \beta_Kx_k))
where the betas are log-ORs. The proposed score makes sense only if it able to beat the predictive power of the multivariate logistic regression. The only way to prove this is to fit a logistic regression model
logit(Prob(MACCE=1))=beta_0+beta_1 score and compare this model to the multivariate regression of Table 3.

Response

We appreciate the reviewer’s thoughtful suggestion. Following his/her comment, we constructed the multivariate logistic regression model and compared its performance with our scoring system. When the C-stats were calculated for each model, no statistically significant difference between the two models was observed (0.665 vs. 0.658 for the scoring system and multivariate logistic regression model, respectively). We believe that our scoring system has an advantage over the traditional logistic regression model, given, its simplicity and relevance of chosen variables in clinical practice.

Accordingly, we have added the following sentence to the revised manuscript (page 16, line 15)

“To assess the predictive power of this model, the proposed performance scoring system was compared with that of the traditional multivariate regression model (covariates selected from variables in Table 3) and only a small difference in C-statistics between them was observed (0.665 vs. 0.658).”

2) Line 8, page 16 "We created a scoring model (J-CTO Extension score) according to the odds ratio". I was not able to understand the rule that has been used to transform the odds ratios into the scores. Please clarify.

Response

We would like to thank the reviewer for the comment. To construct our scoring model, identified variables (from traditional logistic regression analyses) were assigned a weighted integer based on the calculated odds ratio. This method has been validated in previous reports. For example, R. Mehran, et al. demonstrated that this integer scoring system was useful in prediction of contrast induced nephropathy (J Am Coll Cardiol 2004;44:1393-1389). More recently, the National Cardiovascular Data Registry (from the American College of Cardiology) developed risk estimation models using the same method (J Am Coll Cardiol 2010;55:1923-32 and J Am Coll Cardiol 2013;6:790-799). These methods are simple and perhaps user-friendly. We have changed the aforementioned sentence, as follows:

“We created a scoring model (J-CTO extension score) by assigning a weighted integer based on the calculated OR”

3) If I understood correctly (not clear from text), the score is defined by summing the scores in the last column of Table 3. This makes sense if the cumulative effects of hemodialysis, LVEF<35%, in-stent occlusion and DM=1 is additive. However, the multivariate logistic regression of Table 3 clearly tells another story, that the cumulative effect of these factors is nonlinear.

Response

We would like to thank the reviewer for the valuable comments. The reviewer correctly stated that the score is defined by summing the scores listed in Table 3. To address the reviewer’s concern, we have directly calculated the incidence of MACCE and number of the variables group (variable number = 0, 1, 2 and 3 or 4).

Figure A

As demonstrated in Figure A, the association of MACCE incidence and cumulative effect of the variable listed in the score was indeed linear. We have also looked at the distribution of the aforementioned variables among our patients (Figure B). The majority of the patients with the above variables (hemodialysis [HD], in-stent occlusion [ISO], and low LVEF) did not overlap in patients with or without diabetes (DM).

Figure B

Please also note that HD, in stent occlusion, low LVEF, and DM were independently associated with risk of MACCE within our original logistic regression analysis. We have also compared the performance of this inter scoring system with a traditional logistic regression model (in response to Comment #1), and the c-stats were almost equivocal.

Please note that we have added the following sentence in our revised manuscript (line 14 page 23

“The association of the MACCE incidence and the cumulative effect of the variable listed in the score was linear and these variables were independently associated with the risk of MACCE development.”

SPECIFIC ISSUES

1) Please clarify that MACCE means "major adverse cardiac and cerebrovascular event" in the abstract.

Response

According to the reviewer’s point, we have added the sentence “major adverse cardiac and cerebrovascular event” in the Abstract section.

2) The phrase "the area under the curve" appears often in both the abstract and the main text: it should be replaced by "the are under the ROC curve".

Response

Following the reviewer’s suggestion, we have replaced the phrase “the area under the curve” with “the area under the ROC” in the manuscript.

3) page 9, line 15: why "paired" t-test? I guess that the analysis is not based on paired observations. Please check.

Response

We agree with the reviewer. According to the reviewer’s suggestion, we have revised this point from paired to unpaired.

4) From Figures 2 and 3A, it seems that the proposed score is treated as a categorical covariate. Why? Cutting a continuous variable in classes is a unnecessary waste of information and it is not generally recommended.

Response

The reviewer raised an important point which we agree with.

One of the purposes of this study was to assess the risk stratification of CTO-PCI. To assess the risk of each case simpler, we evaluated the four-risk groups.

5) I was not able to understand what figure 3B represents. How were the predicted and the observed risks computed? Please clarify.

Response

We appreciate the reviewer’s important suggestion.

For calibration of this scoring model performance, we represented Figure 3B.

We have divided the validation cohort into 10 groups according to the risk score and calculated the predicted risks by logistic regression analysis. Then, we compared the observed and the predicted risks in each group.

We have revised the correlation between the observed and predicted risks and there was significant correlation between of them (r = 0.77).

According to this result, we have replaced the Figure 4B in the revised manuscript (below).

Figure 4B

6) Although the authors declare that the data are fully available without restriction, there is no data attached. The data should be attached to the paper as supplementary file to allow results reproducibility.

Response

The data and materials used to conduct this research are available to the researchers to reproduce the results or replicate the procedure on request. The procedure does need to follow the Act on the Protection of Personal Information Law (as of May 2017) and the Ethical Guidelines for Medical and Health Research Involving Human Subjects (as of March 2015) in Japan.
